# Recurrent mutation of IGF signalling genes and distinct patterns of genomic rearrangement in osteosarcoma

**DOI:** 10.1038/ncomms15936

**Published:** 2017-06-23

**Authors:** Sam Behjati, Patrick S. Tarpey, Kerstin Haase, Hongtao Ye, Matthew D. Young, Ludmil B. Alexandrov, Sarah J. Farndon, Grace Collord, David C. Wedge, Inigo Martincorena, Susanna L. Cooke, Helen Davies, William Mifsud, Mathias Lidgren, Sancha Martin, Calli Latimer, Mark Maddison, Adam P. Butler, Jon W. Teague, Nischalan Pillay, Adam Shlien, Ultan McDermott, P. Andrew Futreal, Daniel Baumhoer, Olga Zaikova, Bodil Bjerkehagen, Ola Myklebost, M. Fernanda Amary, Roberto Tirabosco, Peter Van Loo, Michael R. Stratton, Adrienne M. Flanagan, Peter J. Campbell

**Affiliations:** 1 Cancer Genome Project, Wellcome Trust Sanger Institute, Wellcome Trust Genome Campus, Hinxton, Cambridgeshire CB10 1SA, UK; 2 Department of Paediatrics, University of Cambridge, Cambridge CB2 0QQ, UK; 3 Corpus Christi College, Cambridge CB2 1RH, UK; 4 The Francis Crick Institute, London NW1 1AT, UK; 5 Department of Histopathology, Royal National Orthopaedic Hospital NHS Trust, Stanmore, Middlesex HA7 4LP, UK; 6 Theoretical Biology and Biophysics (T-6), Los Alamos National Laboratory, Los Alamos, New Mexico 87545, USA; 7 UCL Great Ormond Street Institute of Child Health, London WC1N 1EH, UK; 8 Oxford Big Data Institute and Oxford Centre for Cancer Gene Research, Wellcome Trust Centre for Human Genetics, Roosevelt Drive, Oxford OX3 7BN, UK; 9 University College London Cancer Institute, Huntley Street, London WC1E 6BT, UK; 10 Department of Paediatric Laboratory Medicine, The Hospital for Sick Children, Toronto, Ontario, Canada M5G 1X8; 11 Department of Genomic Medicine, MD Anderson Cancer Center, University of Texas, Houston, Texas 77030, USA; 12 Bone Tumour Reference Centre, Institute of Pathology, University Hospital Basel, University of Basel, Basel 4031, Switzerland; 13 Oslo University Hospital, Oslo 0379, Norway; 14 University of Bergen, Bergen 5020, Norway; 15 Department of Human Genetics, University of Leuven, Leuven B-3000, Belgium; 16 Department of Haematology, University of Cambridge, Hills Road, Cambridge CB2 2XY, UK

## Abstract

Osteosarcoma is a primary malignancy of bone that affects children and adults. Here, we present the largest sequencing study of osteosarcoma to date, comprising 112 childhood and adult tumours encompassing all major histological subtypes. A key finding of our study is the identification of mutations in insulin-like growth factor (IGF) signalling genes in 8/112 (7%) of cases. We validate this observation using fluorescence *in situ* hybridization (FISH) in an additional 87 osteosarcomas, with IGF1 receptor (*IGF1R*) amplification observed in 14% of tumours. These findings may inform patient selection in future trials of IGF1R inhibitors in osteosarcoma. Analysing patterns of mutation, we identify distinct rearrangement profiles including a process characterized by chromothripsis and amplification. This process operates recurrently at discrete genomic regions and generates driver mutations. It may represent an age-independent mutational mechanism that contributes to the development of osteosarcoma in children and adults alike.

## Abstract (web summary)

Osteosarcoma is a primary malignancy of bone that affects children and adults. Here, the authors sequence childhood and adult osteosarcomas, identifying mutations in insulin-like growth factor signalling genes and distinct genomic rearrangement profiles characterized by chromothripsis-amplification.

Since the introduction in the early 1980s of systemic cytotoxic chemotherapy into treatment schedules, the prognosis of osteosarcoma has essentially stalled, with ∼40% of patients having incurable disease^1^. In an effort to advance the understanding and treatment of osteosarcoma, the genomic alterations that underpin this cancer require systematic analysis. To date, the exomes or whole genomes of 110 cases have been analysed, elucidating a number of features of the osteosarcoma genome. Perry *et al*.^2^ found recurrent mutations in the PI3K/mTOR (phosphatidylinositol-3-kinase/mammalian target of rapamycin) pathway in a series of osteosarcomas mainly studied by whole exome sequencing. In a whole genome study of osteosarcoma, structural variants were identified as a major source of driver mutation. Some of these variants occurred in the context of chromothripsis, the shattering of chromosomes resulting in copy number oscillations^3^. Using whole exome sequencing combined with copy number arrays, Kovac *et al*.^4^ described genomic alterations in osteosarcoma indicative of compromised homology-directed DNA repair.

Here, we report the somatic genetic changes of 112 childhood and adult osteosarcomas. Our series encompasses treatment-naive and pretreated osteosarcomas (Supplementary Data 1). Tumours were chosen, based on availability of high-quality tissue, to represent all major subtypes of osteosarcoma. Twelve tumours arose in the context of a genetic predisposition to osteosarcoma (Supplementary Data 1).

DNA from tumours and paired normal tissue was interrogated by exome (*n*=75) or whole genome sequencing (*n*=37). For every tumour copy number variants were derived, either from SNP6 arrays (exome samples) or from whole genome sequencing reads. In addition, where feasible, RNA sequencing was performed (*n*=7). Catalogues of somatic point mutations and structural variants were compiled and analysed using the pipeline of the Cancer Genome Project^5^. The previously established high precision of our mutation calling pipeline was confirmed through resequencing 8% of coding indels and substitutions.

A key finding of our study was potentially actionable somatic alterations in insulin-like growth factor (IGF) signalling genes in a subset of cases. Furthermore, we describe a distinct pattern of genomic rearrangement, chromothripsis amplification, that generates driver mutations across the osteosarcoma genome.

## Results

### Overview of somatic changes in 112 osteosarcomas

The burden of coding indels and substitutions across the 112 tumours varied from 3 to 316 mutations per tumour (median=38; Supplementary Data 1 and 2). The highest coding mutation burden was observed in PD4901a, a tumour with microsatellite instability. Analysis of 146,573 substitutions in the context of their immediate 5′ and 3′ bases identified 9 mutational signatures among 37 whole genomes (Supplementary Data 1 and 3). The most prevalent signatures were 5 and 8 (ref. 6). Signature 5 represents an age-related mutational process. Signature 8 is of unknown origin and has a striking preponderance in osteosarcoma. Examination of the patterns of rearrangement revealed three distinct cytogenetic configurations of the osteosarcoma genome, discussed in more detail later.

### A diverse range of cancer genes operates in osteosarcoma

We defined driver mutations in established cancer genes, considering point mutations (substitutions and indels) and structural variants, that is, amplifications, homozygous deletions and breakpoints that disrupt genes or generate gene fusions. It should be noted that rearrangements not associated with changes in copy number or allelic balance can only be called in samples subjected to whole genome sequencing. Furthermore, the high burden of structural variation observed in osteosarcoma (Supplementary Fig. 1), particularly amplifications, may risk overcalling putative driver mutations. Consequently, we adopted a conservative strategy, restricting our interest to high confidence variants (see Methods).

**Figure 1:**
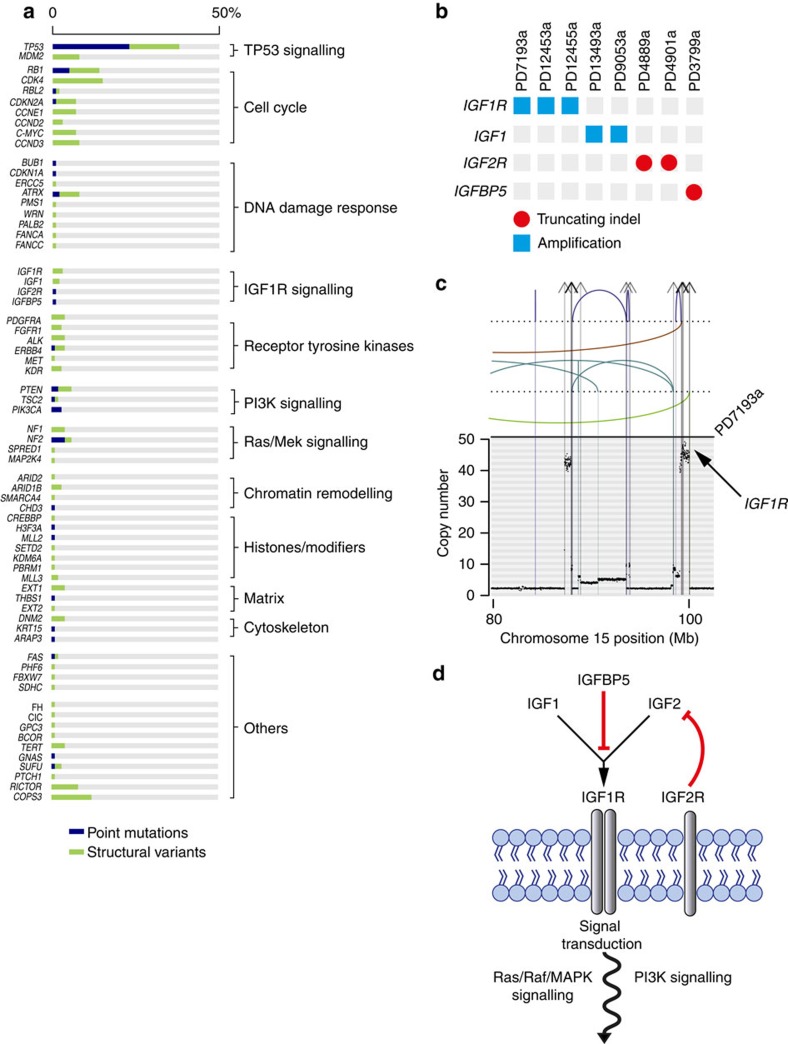
The cancer gene landscape of osteosarcoma. The driver mutations of 112 osteosarcomas are shown in **a**. For each mutated cancer gene the percentage of 112 tumours with at least one driver mutation is shown, subdivided by mutation type. Blue: point mutation (substitutions; indels). Green: structural variant (amplification; homozygous deletion; disruptive breakpoint). (**b**) Driver events that were found in IGF1R signalling genes operative at the level of IGF1R. Blue square: amplification. Red circle: truncating mutation. An example of an amplicon is shown in **c**, found in case PD7193a. The *x* axis shows genomic position in mega bases and the *y* axis shows absolute copy number. Each dot in the plot represents the copy number of a particular genomic position. Lines and arcs: breakpoint with rearrangements coded by colour. Brown: tandem duplication; blue: deletion; green and turquoise: inversion; grey with arrowheads: interchromosomal rearrangement. (**d**) The key components of IGF signalling^7^,^8^,^9^. At the level of the cell membrane, IGF signalling is mediated by IGF1R. IGF2R is a nonsignalling receptor that acts as a negative regulator of IGF1R. A number of circulating binding proteins modulate the function of IGF1R signalling, including IGFBP5 that is thought to inhibit IGF1R. Note that both IGF1 and IGF2 have autocrine, paracrine as well as endocrine sources^7^,^8^,^9^.

Overall, we found a diverse landscape, comprising 67 different cancer genes, with structural variants being the predominant source of mutation (Fig. 1a; driver mutations of individual cases are listed in Supplementary Data 1 and 4). These included 12 cancer genes not previously implicated in osteosarcoma (Table 1). Statistical analyses for nonrandom enrichment in genes and noncoding regulatory sites did not identify novel candidate cancer genes. A number of novel gene fusions were discovered that are predicted to be in-frame, although none was recurrent (Supplementary Data 5). Similarly, no recurrent gene fusions were found in transcriptomic reads.

### Recurrent mutations of IGF signalling genes

We observed recurrent mutations in genes mediating signalling via the IGF receptor (Fig. 1). Mutations in signalling cascades downstream of IGF1 receptor (IGF1R) have previously been found in osteosarcoma^2^. Here, we found mutated genes at the level of the receptor itself in 7% of cases: focal amplification of *IGF1R* (*n*=3) and of *IGF1* (*n*=2); frameshift indels in the recessive cancer genes, *IGF2R* (*n*=2) and *IGFBP5* (*n*=1). These alterations, which were mutually exclusive, would be predicted to result in activation of IGF1R signalling (Fig. 1d)^7^,^8^,^9^. If we include driver mutations downstream of IGF1R affecting the PI3K or Ras/Raf/mitogen-activated protein kinase signalling pathway, perturbed IGF1R signalling may be a driving force in up to 27% of tumours in our series.

Owing to its fundamental role in mediating cellular growth, IGF signalling has long been implicated in the pathogenesis of osteosarcoma^1^,^10^,^11^,^12^,^13^,^14^,^15^. A particular focus of research has been IGF1R that has been targeted in phase I/II clinical trials of IGF1R inhibitors in osteosarcoma^16^,^17^,^18^. Given this prior interest, we assessed *IGF1R* copy number in an extension cohort of 87 cases of childhood and adult osteosarcoma. Using fluorescence *in situ* hybridization (FISH), which allows sensitive and specific examination of individual tumour cells, we found high-level amplification of *IGF1R*, defined as 15 copies or more, in 12/87 (14%) cases (Supplementary Data 6 and Supplementary Fig. 2). These findings justify the reinvigoration of efforts to target IGF1R therapeutically in patients, preselected based on tumour genotype. Given the presence of driver mutations in signalling genes downstream of *IGF1R* in some cases, IGF1R signalling may require targeting at different levels simultaneously to overcome possible intrinsic resistance to IGF1R inhibition alone.

### Patterns of rearrangement define distinct tumour groups

**Figure 2:**
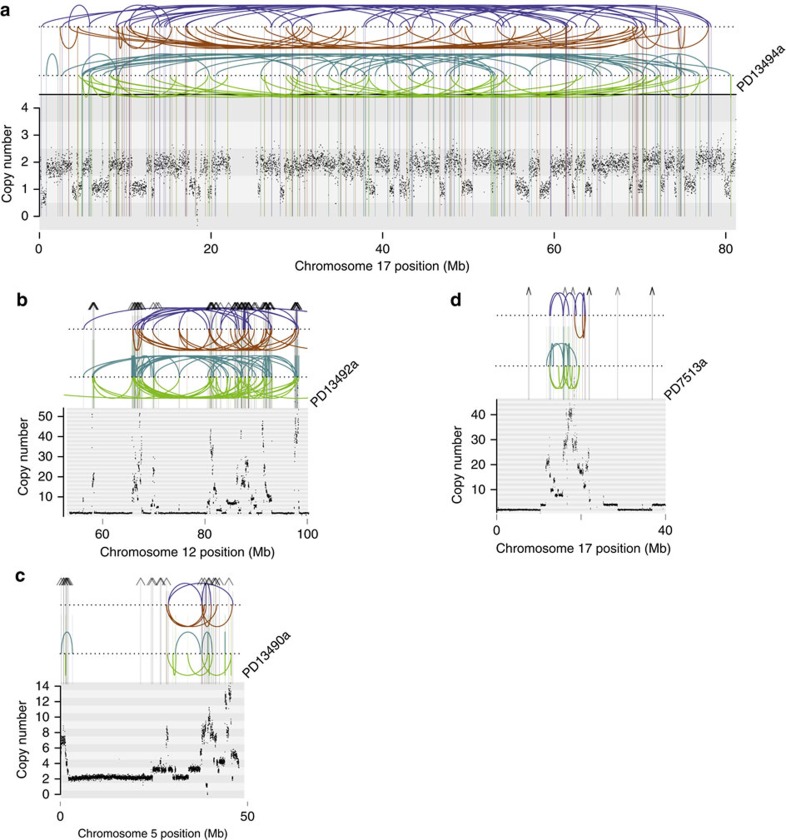
Patterns of rearrangement in osteosarcoma. In **a** the rare case of a tumour is shown in which rearrangements are confined to a single chromosome as a consequence of chromothripsis. This causes multiple driver events in this tumour, that is, loss of heterozygosity (LOH) of *TP53* and *MAP2K4* and disruption of *NF1* by insertion of breakpoints into the gene footprint. (**b**–**d**) Examples of chromothripsis-amplification. The *x* axis shows genomic position in mega bases and the *y* axis shows absolute copy number. Each dot in the plot represents the copy number of a particular genomic position. Lines and arcs: breakpoint with rearrangements coded by colour. Brown: tandem duplication; blue: deletion; green and turquoise: inversion; grey with arrowheads: interchromosomal rearrangement.

Analysis of patterns of rearrangements in 37 tumours revealed three cytogenetic configurations of the osteosarcoma genome. A minority of tumour genomes (4/37) exhibited few or no rearrangements. Given a tumour content of 38% or more in these four osteosarcomas, lack of tumour cells was unlikely to account for the quiet rearrangement profiles. A second profile comprised 11/37 genomes that harboured chromothripsis on one or more chromosomes. A striking and rare example is PD13494a in which chromosome 17 was singularly mutated by chromothripsis with the remaining genome devoid of rearrangements (Fig. 2a). All discernible driver events of this tumour were caused by the disruption on chromosome 17, raising the possibility that PD13494a arose from a single event of chromothripsis. The third profile, seen in 22/37 genomes, was characterized by a distinct copy number pattern of combined chromothripsis and amplification (Fig. 2b–d).

### Chromothripsis amplification generates driver mutations

**Figure 3:**
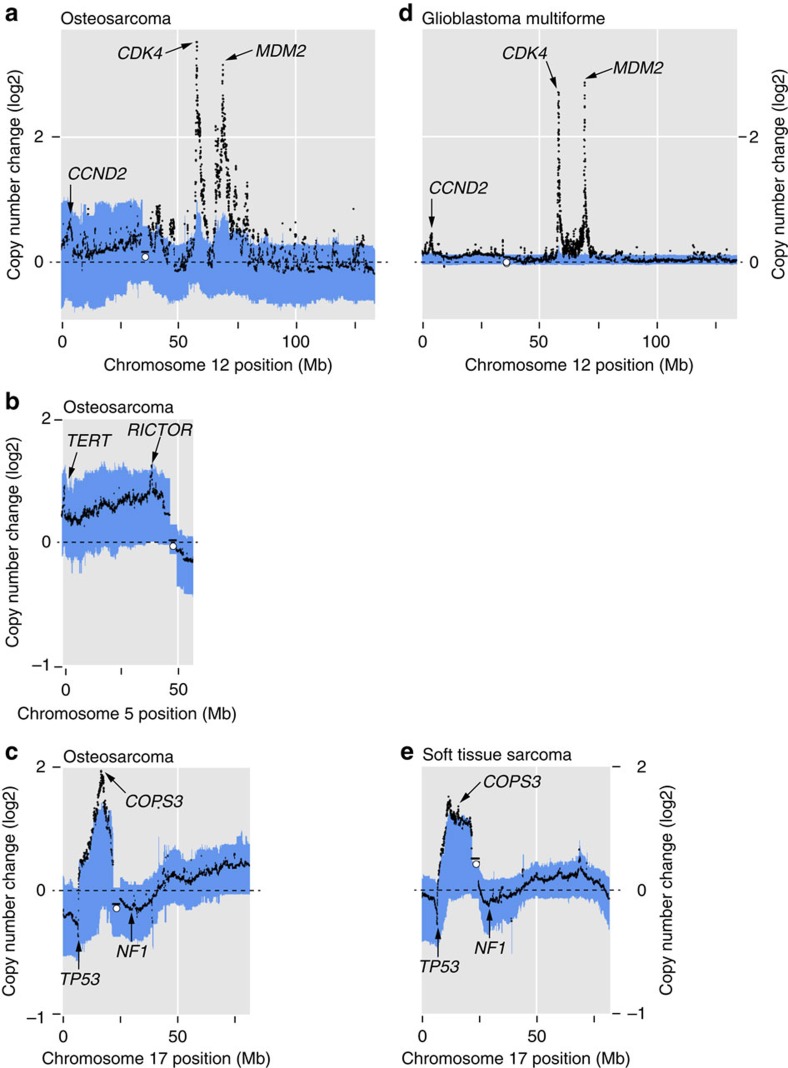
Aggregate copy number profiles. Aggregate copy number profiles of chromosomes 12 (**a**), 5 (**b**) and 17 (**c**) in 112 osteosarcomas are shown in comparison with chromosome 12 of 643 cases of glioblastoma multiforme (**d**) and to chromosome 17 of 267 soft tissue sarcomas (**e**). Using copy number calls from this and other studies, aggregate copy number profiles for each chromosome and tumour type were generated. Blue shaded area highlights the first and third quartiles of copy numbers in each genomic position. Black represents the mean copy number in each genomic position. The *x* axis shows genomic position and the *y* axis shows copy number change (log2). Putative target genes are indicated with arrows.

Chromothripsis amplification affected discrete genomic regions recurrently, including chromosomes 5, 12 and 17 (Fig. 3a–c). Such recurrence may represent chromosomal fragility or be the result of selection for driver events. Detailed annotation of the consequences of rearrangements in these regions across the 37 genomes supports the latter view (Table 2 and Fig. 3). It showed that chromothripsis amplification generated multiple driver events. On chromosome 12, chromothripsis amplification was seen in 6/37 cases, resulting in the co-amplification of *CDK4/MDM2* (Figs 2b and 3a and Table 2). This co-amplicon is well established as a driver event in different types of human cancer including in osteosarcoma. *CDK4/MDM2* co-amplification was predominant in parosteal osteosarcoma, and was also present in other subtypes consistent with previous reports (Supplementary Data 1). Of note, in 2/37 genomes, additional copies of the *CCND2* oncogene were gained in the context of *CDK4/MDM2* amplification on chromosome 12. On chromosome 5, in four cases chromothripsis amplification resulted in gains of the *RICTOR* oncogene combined with copy number gains of *TERT* in 2/37 cases (Figs 2c and 3b, Table 2 and Supplementary Data 7). In five tumours, chromothripsis amplification was present on the short arm of chromosome 17 and the immediate peri-centromeric region (Figs 2d and 3c). Three driver events were generated there: amplification of the *COPS3* oncogene^19^ and disruption of *TP53* and *NF1* by copy number loss or by insertion of disrupting breakpoints into the gene footprint (Fig. 3c and Table 2). Taken together, these findings identify chromothripsis amplification as a mechanism responsible for multiple driver events. Of note, in two tumours chromothripsis amplification co-generated drivers on different chromosomes (Table 2).

### Gene expression in areas of chromothripsis amplification

To further examine the consequences of chromothripsis amplification, we analysed gene expression patterns in regions of recurrent chromothripsis amplification. We found significantly increased variance of gene expression levels in the presence of chromothripsis amplification (*P*=2 × 10^−5^, binomial test; Supplementary Fig. 3). It is therefore conceivable that regions of chromothripsis amplification contain general gene dysregulation in addition to specific driver events.

### A pan-cancer search for chromothripsis amplification

As the chromosome 12 *CDK4/MDM2* co-amplicon is present in many different tumour types, it is conceivable that co-generation of drivers on chromosomes 5 and 17 can also be found in tumours other than osteosarcoma. We therefore analysed the copy number profiles of ∼13,000 tumours. Our finding that the *CDK4/MDM2* co-amplicon was associated with *CCND2* gain was also seen in glioblastoma multiforme (Fig. 3d). The pattern of 17p loss in conjunction with *COPS3* amplification was present in a group of 267 soft tissue sarcomas, particularly in leiomyosarcoma (Fig. 3e). The co-amplification of *TERT* and *RICTOR* was only seen in osteosarcoma.

### Childhood versus adult osteosarcoma

Osteosarcoma is one of few solid cancers with a bimodal age distribution, with the incidence peaking during adolescence and in old age. Our study has not revealed any genomic differences between tumours of different age groups (Supplementary Data 8). The only exception was the burden of substitutions attributable to clockwise mutational processes 1 and 5 that correlates with age (*P*=0.005, Pearson’s product-moment correlation). Chromothripsis amplification was present in patients of all ages, indicating that it is an age-independent rearrangement process.

## Discussion

Sequencing efforts of recent years have repeatedly shown that rare tumours tend to lack genetic diversity and are often driven by highly recurrent, in some cases pathognomonic driver mutations^20^. Osteosarcoma is an exception. Our findings along with previous studies have demonstrated that osteosarcoma exhibits a degree of mutational diversity akin to the most common types of human cancer. The diversity is mainly driven by complex rearrangement processes, chromothripsis and chromothripsis amplification that for unknown reasons predominate in osteosarcoma. This is reflected in the burden of focal amplifications in osteosarcoma that is higher than in any other human cancer studied to date (Supplementary Fig. 1). It is conceivable that rearrangement processes are particularly active in cells of the osteosarcoma lineage, driven intrinsically or by unknown mutagens, thus leading to the apparent enrichment of complex structural variation.

We identified chromothripsis amplification as a distinct pattern of rearrangement that is present in childhood and adult osteosarcoma. If it originated in chromothripsis, it would raise the possibility that formation of multiple drivers might have been seeded in single cellular catastrophes. Consistent with this concept the *MDM2/CDK4* co-amplicon has been studied in detail in liposarcoma and was shown to result from initial chromothripsis, followed by amplification and breakage–fusion–bridge cycles^21^. The same process is likely to operate across the osteosarcoma genome, given the strikingly similar patterns of rearrangement that we observed in regions of chromothripsis-amplification.

We have been able to dissect some of the diversity of osteosarcoma by identifying a subgroup of tumours driven by potentially actionable alterations in IGF1R signalling and by defining distinct patterns of rearrangement. However, the genomes of substantially larger series of tumours, ideally embedded in prospective observational trials, will be required to develop a complete picture of the osteosarcoma genome.

## Methods

### Patient samples

Informed consent was obtained from all subjects and ethical approval obtained from Cambridgeshire 2 Research Ethics Service (reference 09/H0308/165). Norwegian samples were collected by approval from the ethics committee of Southeast Norway (reference S-06133). Collection and use of patient samples were approved by the appropriate institutional review board of each institution.

### Sequencing

Tumour DNA was derived from fresh frozen tissue reviewed by a bone pathologist (A.M.F.). Normal tissue DNA was derived from adjacent normal tissue or blood samples. Whole genome and exome sequencing was performed using the Illumina HiSeq 2000 or 2500 platform^5^, using 100 base paired-end sequencing. For whole genome sequencing we followed the Illumina no-PCR library protocol to construct short insert 500 bp libraries, prepare flowcells and generate clusters. The average coverage of tumours was at least 40 × and of normal DNA at least 30 × . For exome sequencing coding DNA was enriched for using target enrichment by bait capture (Agilent). We aimed to cover 70% of coding regions with at least 30 reads.

### Copy number calling

Copy number calls were derived from SNP6 arrays (exome sample) or whole genome sequences (9 cases) using the ASCAT^22^ or ascatNgs algorithm^23^, respectively. The algorithms were also used to determine tumour content of tumour samples.

### Variant detection

The variant calling pipeline of the Cancer Genome Project, Wellcome Trust Sanger Institute, was used to call somatic mutations^5^. We used the following algorithms with standard settings and no additional prost-processing: CaVEMan for substitutions; the Pindel algorithm for indels; and the BRASS algorithm for rearrangements.

### Variant validation

The high precision of the variant calling pipeline of the Cancer Genome Project, Wellcome Trust Sanger Institute, has been consistently demonstrated in previous experiments^5^,^24^,^25^,^26^,^27^,^28^,^29^. Here, we confirmed this through a dedicated validation experiment. We obtained informative validation reads for 8% of coding mutations, 161 indels and 209 substitutions, from 43 tumours. Of these, ∼90% of substitutions and ∼80% of indels were shown to be true, in keeping with previously published performances of our pipeline. All driver events were further scrutinized and only included in the driver table if they passed manual inspection. As for rearrangements, we only included breakpoints in this data set that had been validated by reconstruction at a base pair resolution.

### Driver analysis of mutations in cancer genes and search for novel drivers

We employed a defined strategy to manually curate driver events from the identified somatic mutations, as previously reported^5^. We only considered variants as potential drivers if they presented in established cancer genes. Mutations in recessive cancer genes were considered if they truncated the gene footprint, that is, truncating substitutions, out-of-frame indels, disruptive rearrangement breakpoints and homozygous deletions. An additional requirement for homozygous deletions was that the deleted segment had to be focal (that is, <1 Mb in size). Mutations in oncogenes were considered driver events if they resided at previously reported canonical hot spots (point mutations) or amplified the intact gene. Amplifications additionally had to be focal (<1 Mb in size) and increase the copy number of oncogenes to a minimum of 5 or 9 copies in diploid and tetraploid samples, respectively. To search for driver variants in novel cancer genes or in noncoding regions, we employed previously developed statistical methods that identify significant enrichment of mutations, taking into account various confounders such as overall mutation burden and local variation in the mutability of the genomic region^5^.

### Extension FISH study

A cohort of 87 further cases of childhood and adult osteosarcoma were examined by FISH for copy number of IGF1R, using previously described methods^30^. In brief, deparaffinized sections were pretreated by a pressure cooking for 5 min and subsequently incubated in pepsin solution at 37 °C for 50 min. Probes were applied to tissue sections and denatured at 72 °C, and followed by hybridization overnight at 37 °C. After hybridization, the sections were washed and mounted by 4′,6-diamidino-2-phenylindole with coverslips.

### Meta-analysis of copy number changes in human cancer

Copy number changes across 13,241 tumours were called. CEL files derived from Affymetrix SNP6 arrays were processed using the PennCNV libraries^31^ to obtain logR and BAF data. The logR was subsequently corrected for GC content to decrease wave artefacts. Copy number profiles for all tumour samples were inferred from the corrected data using the ASCAT computational framework version 2.4.2 (ref. 22). Using bespoke R code, aggregate genome wide copy number profiles for each tumour type were generated, visualizing the first and third quantiles as well as the mean. The tumours included in this analysis were: osteosarcoma (*n*=112; this series); chondrosarcoma (*n*=38 (ref. 32)); chordoma (*n*=38; unpublished data); breast cancers (*n*=1,449; sourced from The Cancer Genome Atlas (TCGA) and International Cancer Genome Consortium (ICGC)^5^); acute myeloid leukaemia (*n*=200; TCGA); adrenocortical carcinoma (*n*=90; TCGA); bladder urothelial carcinoma (*n*=439; TCGA); brain lower grade glioma (*n*=531; TCGA); breast invasive carcinoma (*n*=1,175; TCGA); cervical squamous cell carcinoma and endocervical adenocarcinoma (*n*=307; TCGA); cholangiocarcinoma (*n*=49; TCGA); colon adenocarcinoma (*n*=643; TCGA); oesophageal carcinoma (*n*=192; TCGA); glioblastoma multiforme (*n*=589; TCGA); head and neck squamous cell carcinoma (*n*=590; TCGA); kidney chromophobe cancer (*n*=66; TCGA); renal clear cell carcinoma (*n*=631; TCGA); renal papillary cell carcinoma (*n*=335; TCGA); hepatocellular carcinoma (*n*=415; TCGA); lung adenocarcinoma (*n*=715; TCGA); lung squamous cell carcinoma (*n*=588; TCGA); lymphoid neoplasm diffuse large B-cell lymphoma (*n*=48; TCGA); mesothelioma (*n*=87; TCGA); ovarian serous cystadenocarcinoma (*n*=611; TCGA); pancreatic adenocarcinoma (*n*=191; TCGA); pheochromocytoma and paraganglioma (*n*=186; TCGA); prostate adenocarcinoma (*n*=562; TCGA); rectum adenocarcinoma (*n*=183; TCGA); soft tissue sarcoma (*n*=268; TCGA); cutaneous melanoma (*n*=475; TCGA); stomach adenocarcinoma (*n*=529; TCGA); testicular germ cell tumours (*n*=156; TCGA); thymoma (*n*=126; TCGA); thyroid carcinoma (*n*=543; TCGA); uterine carcinosarcoma (*n*=57; TCGA); uterine corpus endometrial carcinoma (*n*=583; TCGA); and uveal melanoma (*n*=80; TCGA).

### Extraction of substitution signatures

Substitution signatures were extracted using the nonnegative matrix factorization algorithm^6^.

### Analysis of gene expression variance

To assess variance of gene expression levels in areas of chromothripsis amplification, we identified genomic regions of chromothripsis amplification in tumours for which we had RNA sequencing data. For all genes in these regions we measured the variability of expression by calculating the variance of the TPM quartiles. To assess whether gene expression was more variable in samples and regions containing chromothripsis amplification, we compared the variance in TPM quantiles for each region between samples with chromothripsis amplification with those that are copy number neutral. If the variability of gene expression were uncorrelated with the copy number state of the region, we would expect that the median variance for the control samples to be higher than the chromothripsis-amplified samples 50% of the time. We tested this null hypothesis against the alternative hypothesis that the variance is higher in regions of chromothripsis amplification using a binomial test.

### Statistical analyses

Other statistical tests employed are detailed elsewhere in this Methods section or in Figure legends.

### Data availability

The authors declare that all data supporting the findings of this study are available within the article and its Supplementary Information Files or from the corresponding author on reasonable request. Sequencing data have been deposited at the European Genome-Phenome Archive (http://www.ebi.ac.uk/ega/) that is hosted by the European Bioinformatics Institute (accession numbers EGAD00001000107, EGAS00001000196 and EGAD00001000147).

## Additional information

**How to cite this article:** Behjati, S. *et al*. Recurrent mutation of IGF signalling genes and distinct patterns of genomic rearrangement in osteosarcoma. *Nat. Commun.* **8,** 15936 doi: 10.1038/ncomms15936 (2017).

**Publisher’s note:** Springer Nature remains neutral with regard to jurisdictional claims in published maps and institutional affiliations.

## Supplementary Material

Supplementary Information

Supplementary Data 1

Supplementary Data 2

Supplementary Data 3

Supplementary Data 4

Supplementary Data 5

Supplementary Data 6

Supplementary Data 7

Supplementary Data 8

## Figures and Tables

**Table 1:** Cancer genes not previously implicated in osteosarcoma.

**Gene**	**Number of mutated samples (*****n*****=112)**	**Truncating point mutation**	**Disruptive break point**	**Homozygous deletion**	**Amplicon**
*DNM2*	4	0	4	0	0
*ERBB4*	4	1	0	3	0
*TERT*	4	0	0	0	4
*ARID1B*	3	0	3	0	0
*CCND2*	3	0	0	0	3
*IGF1R*	3	0	0	0	3
*RBL2*	3	2	1	0	0
*SUFU*	3	1	2	0	0
*FAS*	2	1	0	1	0
*IGF2R*	2	2	0	0	0
*MLL3*	2	0	2	0	0
*SETD2*	2	1	1	0	0

Listed are mutated cancer genes in our series of 112 osteosarcoma. The number of mutated samples and mutation class for each gene are listed.

**Table 2:** Copy number of putative target genes in areas of chromothripsis amplification.

**Chr ▸**	**5**		**12**			**17**		
**Gene ▸**	***TERT***	***RICTOR***	***CCND2***	***MDM2***	***CDK4***	***TP53***	***COPS3***	***NF1***
Sample ▾								
PD13486a	−	**+3**						
PD7190a	+1	**+6**						
PD9962a	**+5**	**+2**						
PD13490a	**+4**	**+7**				*LOH*	**+13**	*BP*
PD13478a			+1	**+20**	**+22**			
PD13495a			+1	**+5**	−			
PD7401a			**+6**	**+10**	**+8**			
PD7508a			**+2**	**+17**	**+23**			
PD13492a			+1	−	**+15**	*LOH+BP*	**+16**	−
PD13476a						*LOH+BP*	**+16**	*LOH*
PD7513a						*LOH+BP*	**+25**	*LOH*
PD9056a						*LOH+BP*	**+26**	−

BP, breakpoint present within gene footprint; Chr, chromosome; LOH, loss of heterozygosity.

Allele-specific copy number of putative target genes in regions with chromothripsis amplification. For oncogenes (*TERT*, *RICTOR*, *CCND2*, *MDM2*, *CDK4*, *COPS3*) copy number gain of the major allele is shown. For tumour suppressor genes (*TP53*, *NF1*) the minor allele is shown. Numbers preceded by ‘+’ indicate number of extra copies. The bold cells indicate significant copy number gain, the italicized cells indicate significant copy number loss.
