# Supplementary material for: Recurrent mutation of IGF signalling genes and distinct patterns of genomic rearrangement in osteosarcoma
Source: Nat Commun. 2017 Jun 23;8:15936. doi: 10.1038/ncomms15936 (PMC5490007; doi:10.1038/ncomms15936)
Supplement: Supplementary Information [file ncomms15936-s1.pdf]

File Name: Supplementary Information
Description: Supplementary figures.

File Name: Supplementary Data 1
Description: Overview of 112 osteosarcomas.

File Name: Supplementary Data 2
Description: Coding indels and substitutions in 112 osteosarcoma.

File Name: Supplementary Data 3
Description: Somatic indels and substitutions in 37 osteosarcoma genomes.

File Name: Supplementary Data 4
Description: Driver mutations in 112 osteosarcoma.

File Name: Supplementary Data 5
Description: Rearrangements in 37 osteosarcoma genomes.

File Name: Supplementary Data 6
Description: IGF1R FISH in an extension series of 87 osteosarcomas.

File Name: Supplementary Data 7
Description: Copy number changes in 112 osteosarcomas.

File Name: Supplementary Data 8
Description: Association of genomic feature with age.

Supplementary Figure 1.

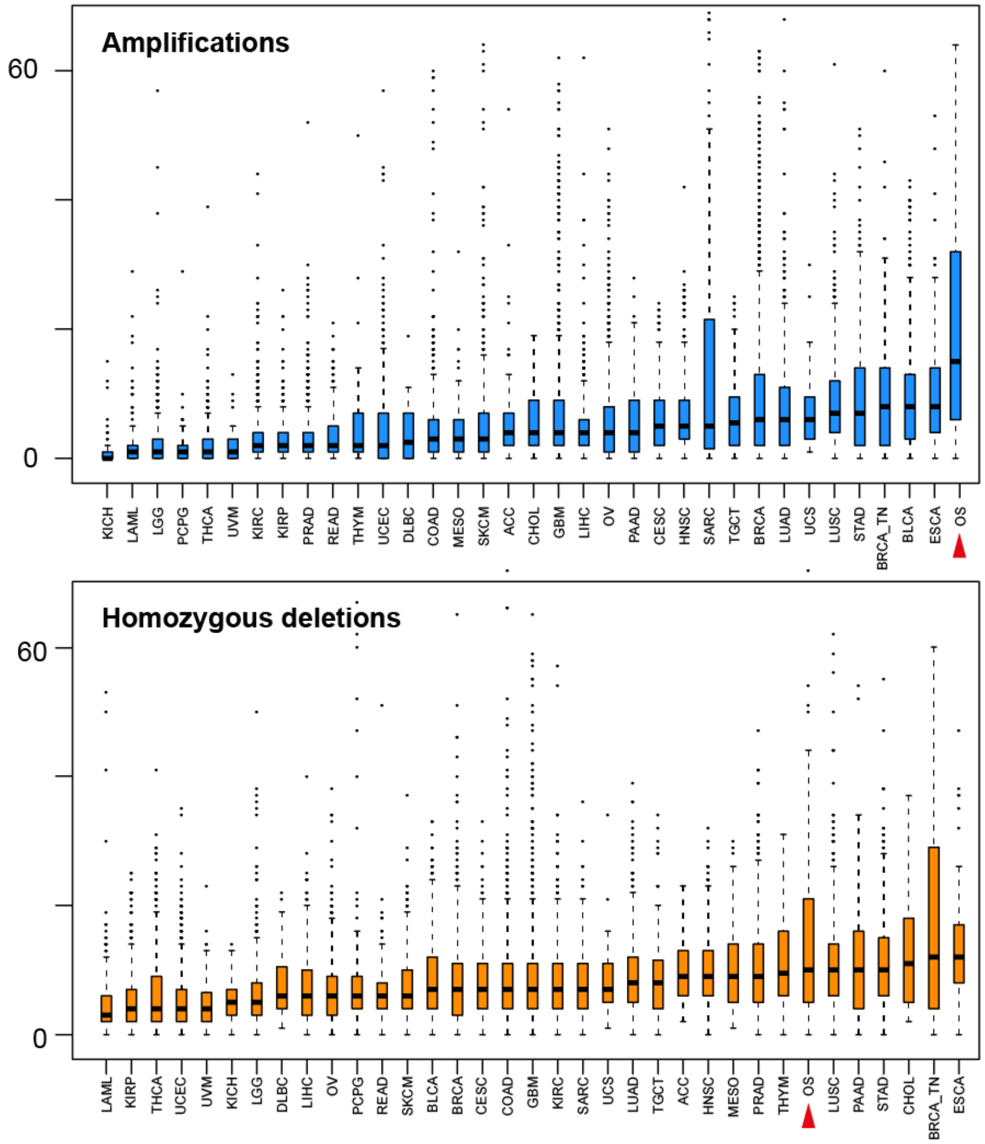

Supplementary Figure 1. Burden of amplifications and homozygous deletions across human cancer. Osteosarcoma display significantly elevated burden of amplifications. Shown is the burden of focal amplifications or focal homozygous deletions in ~13,000 tumours by tumour type. For definitions and source of data see Methods. X-axis: tumour type. Arrowhead highlights osteosarcoma. Y-axis: Absolute burden per sample. Vertical line in boxplots - median. **Abbreviations of tumour types (X-axis):** ACC - Adrenocortical carcinoma; BLCA - Bladder Urothelial Carcinoma; BRCA - Breast invasive carcinoma; BRCA_TN – Triple Hormone Negative Breast invasive carcinoma; CESC - Cervical squamous cell carcinoma and endocervical adenocarcinoma; CHOL – Cholangiocarcinoma; COAD - Colon adenocarcinoma; DLBC - Lymphoid Neoplasm Diffuse Large B-cell Lymphoma; ESCA - Esophageal carcinoma; GBM - Glioblastoma multiforme; HNSC - Head and Neck squamous cell carcinoma; KICH - Kidney Chromophobe; KIRC - Kidney renal clear cell carcinoma; KIRP - Kidney renal papillary cell carcinoma; LAML - Acute Myeloid Leukemia; LGG - Brain Lower Grade Glioma; LIHC - Liver hepatocellular carcinoma; LUAD - Lung adenocarcinoma; LUSC - Lung squamous cell carcinoma; MESO – Mesothelioma; OS – Osteosarcoma; OV - Ovarian serous cystadenocarcinoma; PAAD - Pancreatic adenocarcinoma; PCPG - Pheochromocytoma and Paraganglioma; PRAD - Prostate adenocarcinoma; READ - Rectum adenocarcinoma; SARC – Sarcoma; SKCM - Skin Cutaneous Melanoma; STAD - Stomach adenocarcinoma; TGCT - Testicular Germ Cell Tumors; THCA - Thyroid carcinoma; THYM – Thymoma; UCEC - Uterine Corpus Endometrial Carcinoma; UCS - Uterine Carcinosarcoma; UVM - Uveal Melanoma

Supplementary Figure 2.

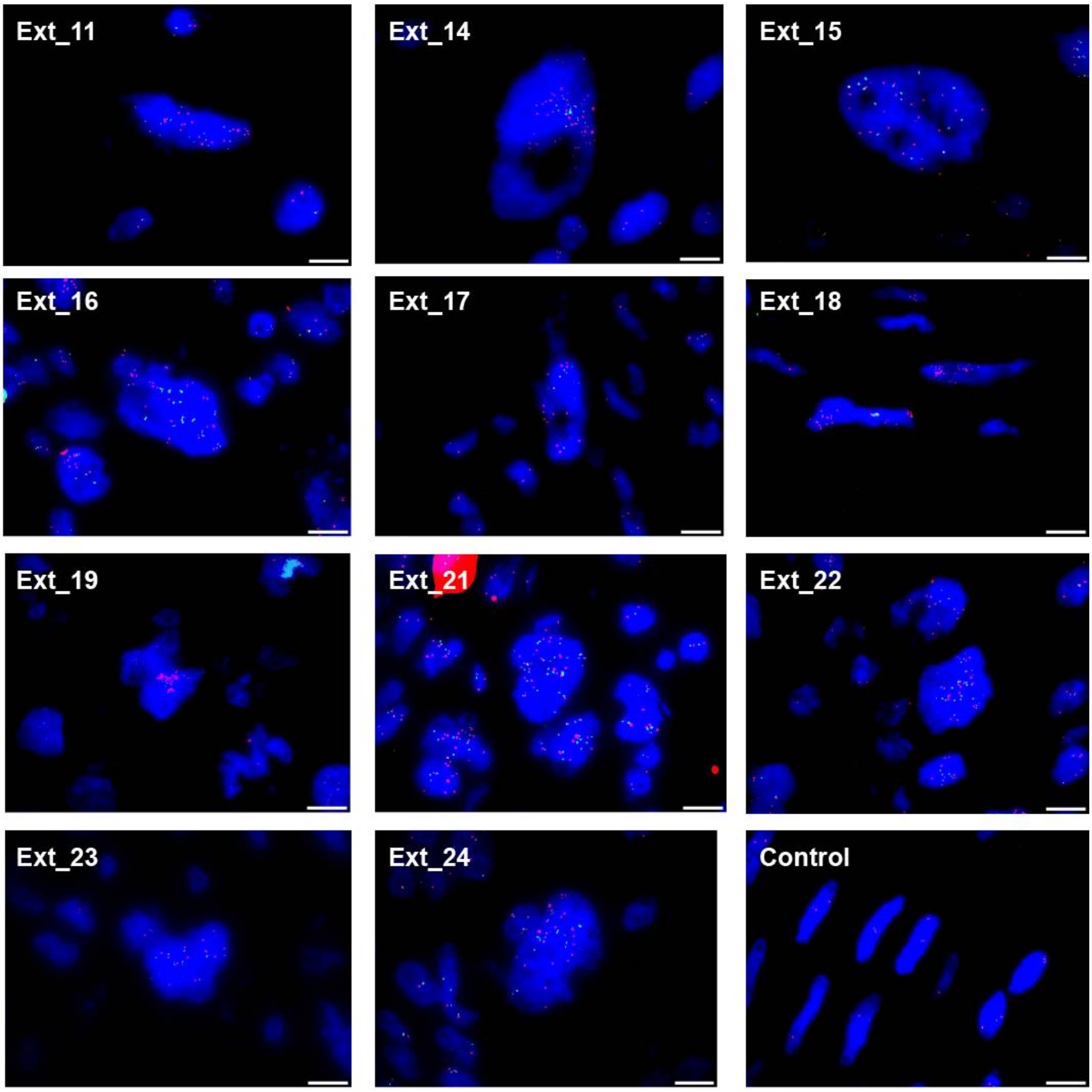

Supplementary Figure 2. IGF1R amplification in extension series. Fluoresecent in situ hybridisation for IGF1R (red) was performed on an extension series of 87 cases. In 12 tumours high level amplification (> 15 copies per cell) of IGF1R was detected. The chromosome (telomere) is labelled with green fluorescent probe. IDs correspond to case numbers as per Supplementary Table 6. Note that in one case with IGF1R amplification (Ext_20) a high quality image could not be obtained. Amplification in this sample was verified by a second observer. Scale bar: 10 µm.

Supplementary Figure 3.

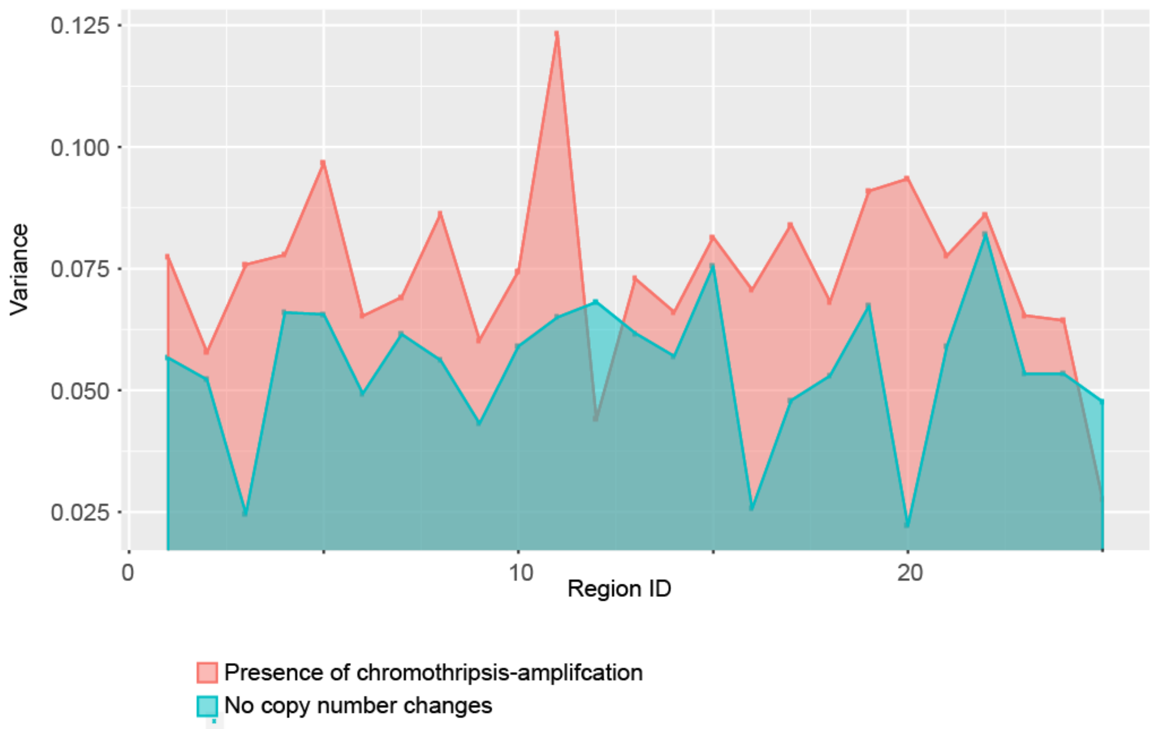

Supplementary Figure 3. Increased diversity of gene expression levels in areas of chromothripsis-amplification. Figure shows the median variance of gene expression (as measured by the variance in TPM quantiles) for each of 25 genomic regions with (green), and without (red) chromothripsis-amplification (red). The variance was significantly increased in the presence of chromothripsis-amplification (p = 2 × 10-5, binomial test).
